# Stride‐to‐Stride Fluctuations and Temporal Patterns of Muscle Activity Exhibit a Stronger Relationship in Running‐Induced Fatigue

**DOI:** 10.1111/sms.70223

**Published:** 2026-02-09

**Authors:** Christos Chalitsios, Vasileios Mylonas, Nick Stergiou, Thomas Nikodelis

**Affiliations:** ^1^ AUTH Biomechanics Laboratory, School of Physical Education and Sports Science, Aristotle University of Thessaloniki Thessaloniki Greece; ^2^ Department of Biomechanics and Center for Research in Human Movement Variability, Division of Biomechanics and Research Development University of Nebraska at Omaha Omaha Nebraska USA

**Keywords:** biomechanics, complexity, gait, locomotion, variability

## Abstract

During gait, the temporal patterns of stride‐to‐stride fluctuations and muscle activation exhibit similar responses. This study examined if increasing task demands such as fatigued high intensity running affects this relationship. Eleven experienced runners completed two 400‐m runs (*R*_1_, *R*_2_) with a 3‐min break to induce fatigue. Stride Time Intervals (STI) were measured using inertial sensors (IMUs), while Inter‐Muscle Peak Intervals (IMPI) were derived from the electromyographic (EMG) activity of the Vastus Lateralis and Gastrocnemius Medialis. We calculated the Hurst exponent and coefficient of variation (CV) for all time series. The association (matching) between STI and IMPI metrics was analyzed using Pearson's correlation. To assess fatigue status, we monitored muscle oxygen saturation (SmO_2_) and EMG frequency characteristics. Hurst exponent was estimated from the Hurst—Kolmogorov process which is proposed as suitable for short time series. Baseline SmO_2_ immediately prior to the second run was significantly lower for *R*_2_ (*p* < 0.001). This was supported by EMG fatigue signatures: median frequency decreased and root mean square increased in both muscles. Significantly higher Hurst values were observed for all time series, whereas CV increased significantly only for the STI and Vastus Lateralis IMPI in *R*_2_ (*p* < 0.05). The STI‐IMPI correlations (for CV and Hurst) increased significantly in *R*_2_ for both VL and GM (*r* = 0.66 to 0.96). The changes in EMG characteristics indicate less flexible neuromuscular control and possibly provide a physiological explanation for the increased Hurst exponent (persistency). Fatigue strengthened the coupling between stride‐to‐stride fluctuations and muscle activation. This suggests high‐intensity fatigue causes the locomotor system to reorganize into a more tightly coupled, less flexible state.

## 1 Introduction

Over the past decades, there has been increasing interest in the investigation of the temporal structure of variability in biological signals using non‐linear analytical methods to answer questions regarding control of movement within the context of specific organismic and environmental constraints [1, 2]. Such investigations have been conducted in several subsystems such as cardiac rhythms [3], blood pressure [4], electromyographic (EMG) activity [5, 6], stride time intervals [7, 8], and postural sway [9]. Additionally, the relationship among physiological subsystems regarding the temporal structure of variability is often identified [5, 6, 10]. During gait, for example, the temporal structure of stride time intervals (STI) has been shown to correlate with muscle activation patterns that are imprinted in the time variations between muscle activation peak intervals (IMPI) of the gastrocnemius muscle [5] as well as with the time intervals between consecutive heartbeats [10]. While subsystem variability coupling may seem straightforward and intuitively compelling, substantial evidence also suggests that under optimal conditions, subsystems remain loosely coupled [10, 11, 12]. Strength of cardio‐locomotor coupling increases in demanding compared to nondemanding conditions and in older compared to younger adults [11, 12, 13]. Under demanding and overly constrained conditions, therefore, the locomotor system may no longer operate in an optimal state but instead reorganize into a strongly coupled and less flexible behavior [14].

Complexity in human physiology refers to the structured variability observed in biological outcomes over time. This structured variability in healthy systems demonstrates a degree of persistence, resulting in a correlation between consecutive fluctuations. For example, in the context of gait variability, fast steps tend to be followed by fast ones, and slow steps tend to be followed by slow ones. This pattern is present in several healthy human subsystems, as literature from several disciplines has shown that many apparently “noisy” phenomena are the result of nonlinear interactions leading to complex patterns in variability [1, 7]. Age and pathology can lead to loss of physiological complexity that is characterized by alterations in the temporal structure of variability [2, 7, 15, 16]. As a result, the variability becomes too predictable (rigid) or too random. This phenomenon is described in the Optimal Movement Variability Hypothesis (OMVH), according to which fluctuations in stride‐to‐stride (among other repeated movements) time intervals display an “optimal” structure that is neither random nor predictable [2, 14, 17]. This temporal structure of movement variability reflects the self‐organization of human movement given the environmental, task, and organismic constraints [14, 18, 19]. These constraints can influence both the complexity of individual outcomes (e.g., stride‐to‐stride variability) and the interactions among subsystems. There is also evidence suggesting that relationships between subsystems are affected by task constraints, manipulation of which (walking faster than preferred walking speed) leads to increased coupling between cardiac and locomotor complexity [10]. Furthermore, coupling is stronger in older adults compared to younger adults, suggesting that organismic constraints can also affect how different subsystems interact [10, 11, 13]. In this context, coupling denotes the extent to which the variability patterns of two physiological systems synchronize or interdepend over time. Finally, manipulation of environmental constraints through provision of external cues can also increase the coupling between subsystems [10]. While this increase may appear beneficial, excessive coupling may reflect reduced flexibility in the system. Optimal motor control often requires segregation of the system's components, and too much synchrony may indicate rigidity rather than adaptability [14].

The optimal structure that is typically observed in stride‐to‐stride variability of healthy individuals during walking is also observed during running [20]. Loss of complexity in running gait has been linked to previous injuries [21] and fatigue [21, 22, 23]. In running gait, exercise‐induced fatigue causes adaptations of several organismic mechanisms to meet the task‐specific demands significantly affecting musculoskeletal running mechanics [24, 25]. Adverse effects on neuromuscular function can lead to a reduction in mechanical energy transfer during the stretch–shortening cycle and muscle reaction times [26]. As physiological stress accumulates, muscular efficiency declines, leading to altered running biomechanics. Nonetheless, these modifications may not be inherently harmful; rather, they may signify essential compensatory mechanisms for maintaining movement during fatigue [27]. These biomechanical adaptations are frequently manifested in the temporal structure of stride‐to‐stride intervals, which tend to exhibit increased randomness as fatigue intensifies [21, 22], and are commonly accompanied by increases in the overall magnitude of variability, such as greater standard deviation and coefficient of variation (CV) in step parameters [28, 29].

Physiologically, these mechanical adaptations are underpinned by distinct neuromuscular adjustments detectable via surface electromyography (EMG) [30, 31]. As fatigue accumulates and muscle fiber conduction velocity slows, the electrical signal typically exhibits ‘spectral compression’—a shift towards lower frequencies—often accompanied by a compensatory increase in signal amplitude (RMS) to maintain force output [30, 31, 32]. Monitoring these spectral and amplitude characteristics provides a window into the rigidity of the neuromuscular control strategy adopted to cope with high‐intensity demand.

It is thus evident that the literature has provided a wealth of knowledge with respect to the variability of different subsystems. However, there is also a clear knowledge gap regarding how these subsystems are interconnected or coupled, and whether such interconnections are affected by increased task demands, such as fatigue. Therefore, we sought to examine how STI correlate with IMPI patterns during high‐intensity running‐induced fatigue. To this end, we selected a submaximal short (400‐m) run, which is used regularly in high‐intensity interval training sessions and considered a physically very demanding locomotor task, as it leads to high levels of physiological stress and physical exertion [33].

The purpose of the present paper was to investigate the effect of fatigue on STI and IMPI temporal structure and magnitude of variability as well as on their association in trained track and field athletes. Specifically, we investigated whether introducing higher physical demands in two consecutive (*R*_1_, *R*_2_) submaximal (400‐m) running trials would induce changes in the Hurst exponents (*H*) and CV values of STI and IMPI as well as the correlation between the two. As a preliminary hypothesis, we tested whether the protocol effectively induced fatigue, expecting lower baseline muscle oxygenation (SmO_2_) before *R*_2_ compared with *R*_1_, accompanied by distinct neuromuscular alterations—specifically, an increase in EMG signal amplitude (RMS) and a concurrent decrease in the spectral characteristics (Median Frequency). We hypothesized that the second run (*R*_2_), performed under residual fatigue, would show reduced persistency (a measure of the signal's long‐term memory/correlation) and increased magnitude of variability compared to the first run (*R*_1_). Finally, we expected that the relationship between STI and IMPI (both in terms of the structure and magnitude of variability) would be stronger in *R*_2_, reflecting increased matching between subsystems under fatigue.

## 2 Materials and Methods

### 2.1 Participants

Eleven adolescent competitive runners (6 male and 5 female) successfully completed the protocol after providing written informed consent. All runners were healthy and free of any neuromuscular or musculoskeletal problems. They were required to have at least 3 years of regular training and at least 2 years of competing in national championships at distances between 800 and 3000 m. See Table 1 for participants' characteristics. The research was approved by the Ethics Committee of the School of Physical Education and Sport Science at Thessaloniki (ΕC‐12/18‐5‐2020).

**TABLE 1:** Characteristics and performance of the participants during the test (mean ± SD).

Characteristic	Males (*n* = 6)	Females (*n* = 5)
Age (years)	16.9 ± 0.9	16.1 ± 0.7
Weight (kg)	68.1 ± 5.3	57.2 ± 3.3
Height (m)	1.78 ± 0.06	1.63 ± 0.05
Velocity (m/s)	6.21 ± 0.13 (*R*_1_) 6.34 ± 0.08 (*R*_2_)	5.48 ± 0.11 (*R*_1_) 5.50 ± 0.05 (*R*_2_)

### 2.2 Experimental Setup

Biomechanical and physiological data were collected in a looping outdoor track. To minimize variation in ambient conditions, testing of all participants was completed within 4 consecutive days between 17.00 and 20.00. Air temperature and humidity were 18.4°C–21.2°C and 61.3%–72.7%, respectively. Participants were familiarized with the equipment 1 day prior to the experiment, during which they performed a brief 5‐min run at an effortless pace while wearing all the measurement devices. On the testing day, the participants were asked not to eat anything during the 3 h preceding the test. They began with a light running warm‐up at a self‐paced speed for 10 min, followed by 5 min of dynamic stretching. Participants wore their preferred running spikes during the test. The main task was two 400‐m submaximal runs at 80% of their most recent competition performance with a 3‐min rest between them. Speed was recorded using a GPS unit (Samsung Galaxy S9) sampling at 10 Hz, which was strapped to the upper arm of each participant and the target velocity (~80% of maximal effort) was calculated based on recent competition times provided by the national federation records. For each participant, the duration of their most recent four‐hundred‐meter competition was converted into an average velocity. The target speed was subsequently set at 80% of this calculated value. We selected this running task to investigate motor control and variability under demanding conditions—a topic that has received limited attention within the movement complexity literature, mainly because of methodological constraints. This rest interval was selected for the assessment of the effects of fatigue since it will not allow for full recovery, and there will be residual fatigue effects in the second test. To verify the induction of fatigue, muscle oxygenation (SmO_2_) was monitored continuously (starting 3 min before *R*_1_ until 5 min after *R*_2_) using a wireless NIRS sensor (Moxy Monitor, Fortiori Design LLC, Hutchinson, MN) tested for its validity and reliability [34]. The sensor was fixed to the right rectus femoris with adhesive tape and a light shield to prevent signal contamination. Data were acquired at 2 Hz via the VO2 Master Manager app. To determine recovery status, we calculated the 10‐s average SmO_2_ immediately before the start of each run (SmO_2_pre10s). This metric allowed us to assess whether the rest interval was sufficient; a significant drop in the *R*_2_ baseline compared to *R*_1_ would confirm residual fatigue. For the analysis of the running bouts themselves, SmO_2_ data were averaged into 10‐s bins, starting from the moment steady speed was achieved (approx. 8–10 m after onset).

STI intervals were quantified using a wireless Bluetooth, 9‐degree‐of‐freedom IMU (K‐Move, K‐Invent, Biomechanique, Montpellier, France) operating at 1000‐Hz sampling frequency. Only vertical linear acceleration from the IMU was used for further analysis. The minimum detectable step (resolution) of the accelerometer sensor was 4 mg/LSB (least significant bit), and the maximum detectable value was 16 g. The IMU sensor was fixed to the foot dorsum on the participants' right leg. The foot‐mounted placement was chosen to maximize the detection accuracy of initial contact events, as the unattenuated impact shock at this location provides the distinct vertical acceleration peak required for precise temporal analysis. Inter‐muscle activation intervals were assessed with two wireless electromyography (EMG) sensors (K‐Myo, K‐Invent, Biomechanique, Montpellier, France) with the sampling rate set at 1000 Hz, an input impedance > 1 GΩ < 2 μV RMS noise, and a bandwidth of DC‐500 Hz. The first sensor was fixed in the Vastus Lateralis (VL) and the second one on the Gastrocnemius Medialis (GM). These muscles were selected as they represent the primary agonists for the knee extension (impact/loading) and plantar flexion (propulsion) phases of the running gait, allowing for the assessment of proximal‐distal coupling. Impedance was minimized by shaving and cleaning the skin in the relevant area with an alcohol solution. Following recommendations from consensus papers in EMG literature [35, 36], disposable pre‐gelled Ag/AgCl electrodes with an inter‐electrode distance of 20 mm were placed. The sensors were securely fixed with tape.

### 2.3 Data Analysis

**FIGURE 1:**
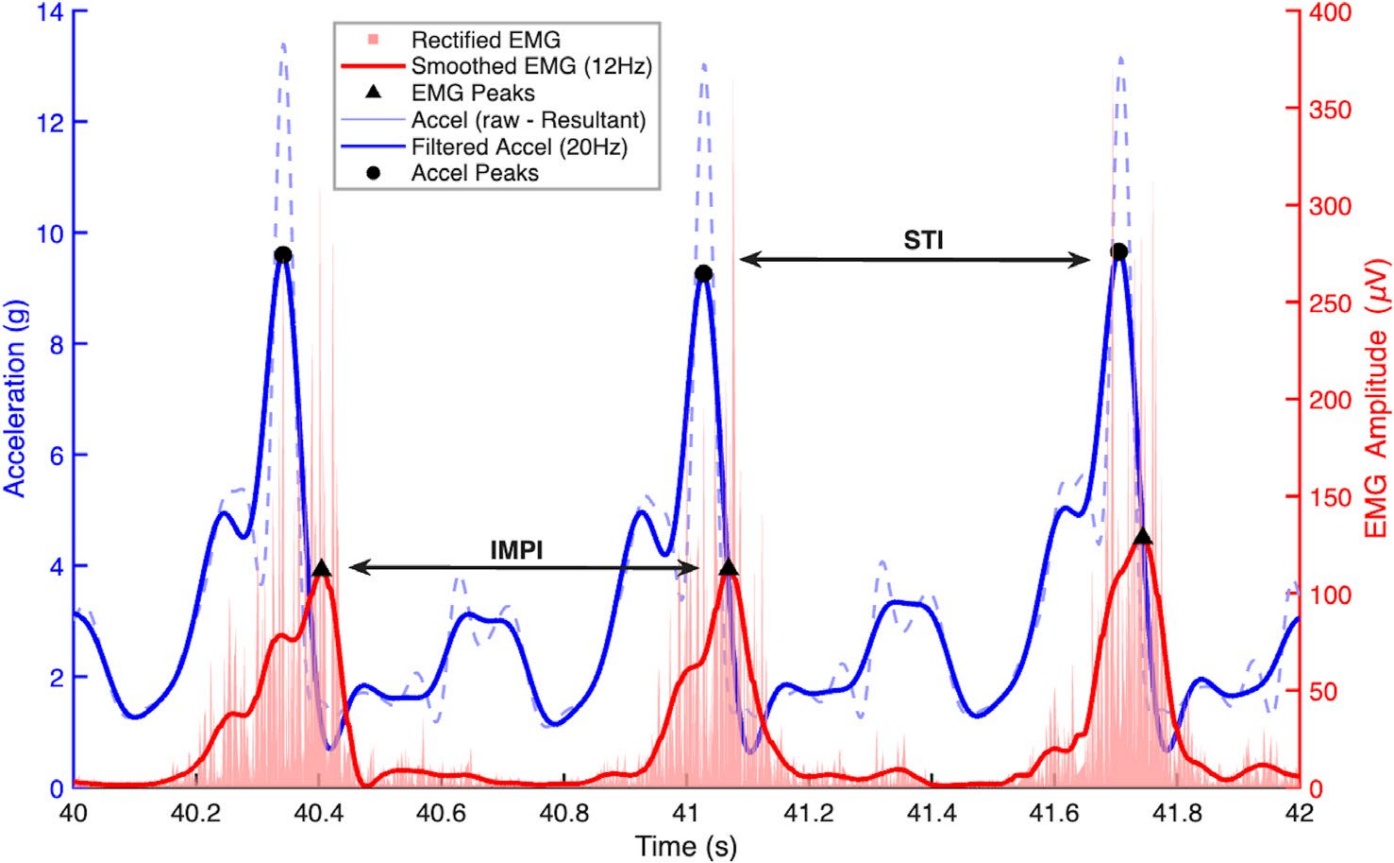
Graphical representation of STI and IMPI calculation from a portion of the IMU and EMG (VL) signals.

For analysis purposes, the first 10 strides were removed to reduce any effects from the acceleration phase although the runners started the test with an incoming speed (flying start). A 4th‐order, zero‐lag low‐pass Butterworth filter with a cutoff frequency of 20 Hz was applied to the accelerometer signal. Filtering cutoff frequency was defined based on residual analysis. A custom MATLAB (2023b, Natick, Massachusetts, MathWorks) script was used to determine STI, identified as the time difference between two consecutive peaks in the accelerometer data (foot strikes) of the same foot. Peaks were identified using the findpeaks function with a minimum peak distance set to 250 ms to prevent the detection of multiple peaks within a single gait cycle. The first 10 strides were removed to strictly ensure the analysis of steady‐state mechanics, a decision verified by the visual inspection of the STI time series which showed stabilization of the variance after this initial period. Raw EMG signals were band‐pass filtered (20–500 Hz), full‐wave rectified, and smoothed with a zero‐lag low‐pass filter (12 Hz, 4th order Butterworth), following the 2017 recommendations from the International Society of Electrophysiology and Kinesiology (https://isek.org/emg‐standards/). Afterwards, the peak maximum from each gait cycle was found, and the time difference between two consecutive peaks was determined as the IMPI. For every signal, visual inspection of the peaks' identification was conducted both on the acceleration signal and on the STI time series to ensure robustness in the detection (Figure 1). To provide complementary evidence of neuromuscular fatigue [32], the raw EMG signals from the VL and GM were further analyzed by segmenting them into “active periods.” These periods were defined using the smoothed EMG envelope and the previously identified peaks; each burst was demarcated by tracing forward and backward from its peak to the points where the amplitude fell to 20% of that peak's value. For each resulting “active period,” two distinct parameters were calculated. First, the Root Mean Square (RMS) was computed from the rectified, band‐pass filtered (20–500 Hz) signal to assess signal amplitude. Second, the Median Frequency (MDF) was computed from the Power Spectral Density (PSD) of the corresponding raw, non‐rectified, band‐pass filtered (20–500 Hz) signal. This process yielded a distribution of RMS values and a distribution of MDF values (one per burst) for each run. From these distributions, three metrics were derived for statistical comparison between *R*_1_ and *R*_2_: the mean of RMS, the mean of MDF, and the Interquartile Range (IQR) of the MDF distribution. The Hurst exponent and the CV were calculated for each STI and IMPI time series. The Hurst exponent was used as a measure of the complexity of the time series, while the CV was used as a measure of the magnitude of variability.

Detrended Fluctuation Analysis (DFA) is the predominant method for assessing stride‐to‐stride variations in gait literature and is used to estimate the H throughout a time series. The Hurst exponent reveals the degree of persistency (0.5 < H < 1.0; large values in the time series are typically followed by large values and vice versa), suggesting a presence of “memory” in the series, where the future values are influenced by past values. The time series could exhibit anti‐persistency (0 < H < 0.5; large values in the time series are usually followed by small values and vice versa), exhibiting “mean‐reverting” behavior. Recent advancements in nonlinear analysis methods now make it possible to examine the complexity of human movement even in short time series [37, 38, 39, 40]. This method makes it feasible to assess complexity in high‐intensity, short‐duration tasks like 400‐m runs. We selected a Bayesian approach to estimate the Hurst exponent using the Hurst‐Kolmogorov (HK) methodology, as outlined by Tyralis and Koutsoyiannis [40]. This decision was influenced by the DFA algorithm's theoretical limitations in analyzing short time series, as noted in the literature [37, 38, 39], where authors also provided comparative validity for various lengths of time series. We chose the HK approach because it is effective for shorter time series [37, 38, 39], unlike methods such as DFA, which require longer time series data (*n* > 600) to accurately estimate the H‐exponent; otherwise, they may introduce a positive bias in their central tendency and result in large dispersions [37, 38]. Finally, from the sampled posterior distribution of the Hurst exponent, we used the median values of the distribution as a point estimate of the Hurst exponent. Time series were analyzed with the HK method using the R function *inferH*() from the package “HKprocess” [41]. The function *inferH*() has two inputs: the time series, *x*_*N*_, and the size of the simulated sample from the posterior distribution of the Hurst exponent, *n*.

### 2.4 Statistical Analysis

All statistical analyses were performed using *JASP (Version 0.95.4)* with all levels of significance set a priori to 0.05. All variables were tested for normality using the Shapiro–Wilk test. To compare all *R*_1_ and *R*_2_ paired data, the normality of the difference scores was first assessed using the Shapiro–Wilk test. For normally distributed data, paired‐samples *t*‐tests were conducted. If the assumption of normality was violated, the non‐parametric Wilcoxon signed‐rank test was applied. This procedure was used to examine differences in running velocities, SmO_2_pre_10s_ (%), all STI and IMPI metrics (CV and Hurst), and all EMG‐derived fatigue metrics (RMS, MDF, and IQR of MDF). Effect sizes were estimated with Cohen's *d* for the parametric test and with the rank‐biserial correlation coefficient (*r*_b_) for the nonparametric Wilcoxon test. The *r*_b_ was interpreted as following: (1) 0.125 < *r*_b_ < 0.304 as a small effect, (2) 0.304 < *r*_b_ < 0.511 as a medium effect, and (3) *r*_b_ > 0.511 as a large effect [42]. The association between the Hurst exponent and CV for STI and IMPI was assessed using Pearson's correlation coefficient.

## 3 Results

Descriptive statistics for the examined variables are presented in Table 2. The average running velocity between the two runs was not statistically different (*p* = 0.19) between *R*_1_ (5.87 ± 0.39 m/s) and *R*_2_ (5.95 ± 0.44 m/s). In contrast, SmO_2_pre_10s_ (%) prior to exercise differed significantly between R_1_ (69.56 ± 5.61) and *R*_2_ (61.36 ± 7.55), *t*(10) = 5.21, *p* = 0.0003, Cohen's *d* = −1.57.

**TABLE 2:** Descriptive statistics of the measured neuromuscular markers: Hurst Exponent, CV, and Spectral Fatigue Indicators for Lower Limb Muscles.

	Muscle/type	*R*_1_	*R*_2_
Stride Time Intervals			
Hurst Exponent		0.76 ± 0.12	0.89 ± 0.11
CV (%)		3.43 ± 1.15	5.72 ± 2.13
Inter‐Muscle Peak Intervals			
Hurst Exponent	VL	0.67 ± 0.15	0.85 ± 0.12
	GM	0.62 ± 0.09	0.72 ± 0.15
CV (%)	VL	4.09 ± 0.86	6.49 ± 2.01
	GM	5.19 ± 1.23	6.67 ± 2.21
Fatigue Indicators			
Median Freq (Hz)*	VL	70.92 ± 6.57	69.08 ± 6.32
	GM	122.8 [18.99]	109.7 [22.04]
RMS (μV)†	VL	335.0 [122.8]	398.0 [124.5]
	GM	312.5 [51.3]	375.6 [135.7]
IQR‐MDF (Hz)†	GM	83.43 [11.01]	80.55 [11.97]

* Presented as Mean ± SD.

^†^ Presented as Median [IQR].

**FIGURE 2:**
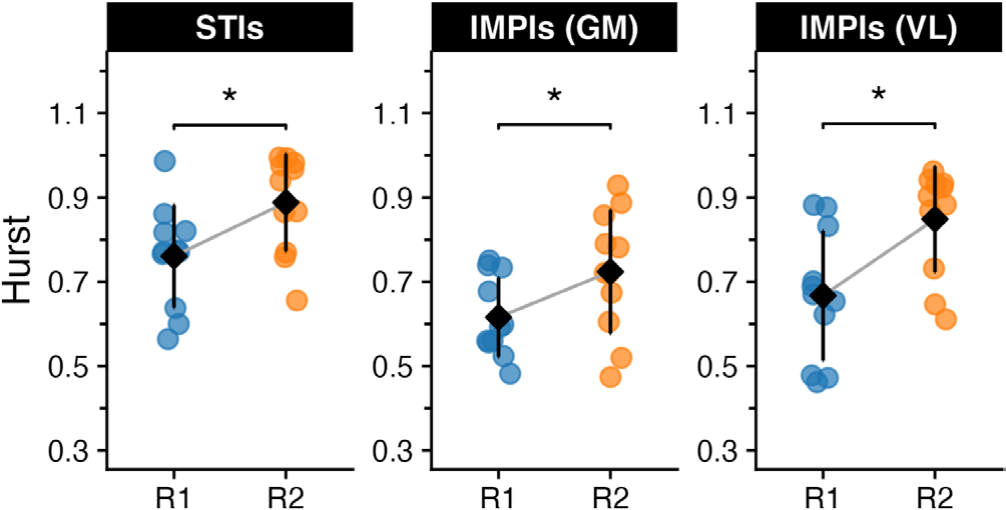
Distributions of the Hurst exponent for the STI and IMPI across different test runs, along with the mean and sd values for each.

There was a statistically significant change in VL MDF from *R*_1_ (70.92 ± 6.57 Hz) to *R*_2_ (69.08 ± 6.32 Hz), *t*(10) = 2.95, *p* = 0.014, Cohen's *d* = 0.89. A Wilcoxon signed‐rank test revealed that the MDF of the GM was significantly lower in *R*_2_ (median = 109.7 Hz, IQR = 22.04 Hz) compared to *R*_1_ (median = 122.8 Hz, IQR = 18.99 Hz), *Z* = 2.13, *p* = 0.032 and a large effect size (*𝑟*_𝑏_ = 0.73). The RMS changes also significantly for the VL between *R*_1_ median = 335 μV, IQR = 122.8 μV and *R*_2_ (median = 398 μV, IQR = 124.5 μV) with *Z* = −2.85, *p* = 0.0019 and a large effect size (*𝑟*_𝑏_ = −0.97). Same was true for GM with *R*_1_ (median = 312.5 μV, IQR = 51.26 μV) and *R*_2_ (median = 375.6 μV, IQR = 135.7 μV) with *Z* = −2.05, *p* = 0.039 and a large effect size (*𝑟*_𝑏_ = −0.7). The MDF‐IQR was found to differ significantly only in the GM with *Z* = 2.84, *p* = 0.0015 and a large effect size (*𝑟*_𝑏_ = 0.97) with *R*_1_ (median = 83.43 Hz, IQR = 11.01 Hz) having higher values compared to *R*_2_ (median = 80.55 Hz, IQR = 11.97 Hz). The MDF‐IQR for VL was not statistically significant (*p* = 0.32) between *R*_1_ (median = 47.04 Hz, IQR = 8.9 Hz) and *R*_2_ (median = 44.32 Hz, IQR = 4.37 Hz). Significant differences were found in the Hurst exponent values for STI (Figure 2), with higher values in *R*_2_ (0.89 ± 0.11) compared to *R*_1_ (0.76 ± 0.12), *t*(10) = −5.52, *p* = 0.0002, and a Cohen's *d* = −1.66. A parallel pattern emerged for the IMPI of the VL muscle (Figure 2), where *R*_2_ exhibited greater Hurst exponent values (0.85 ± 0.12) than *R*_1_ (0.67 ± 0.15) with *t*(10) = −5.65, *p* = 0.0002, Cohen's *d* = −1.7. Similar were the results for the GM muscle with *R*_2_ having greater Hurst exponent values (0.72 ± 0.16) than *R*_1_ (0.62 ± 0.1) with *t*(10) = −2.44, *p* = 0.034, Cohen's *d* = −0.73.

**FIGURE 3:**
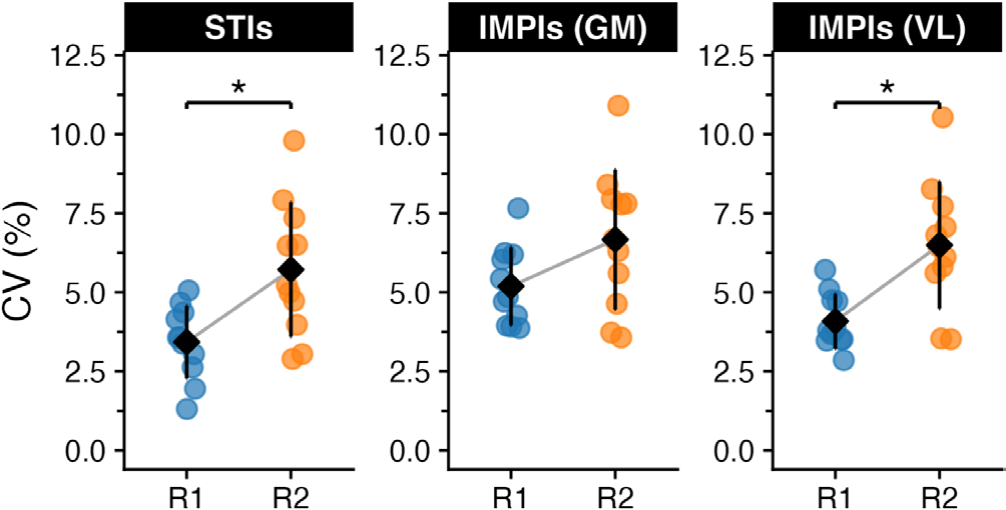
Distributions of the CV for the STI and IMPI across different test runs, along with the mean values for each.

CV for STI was significantly higher in *R*_2_ (5.72% ± 2.13%) compared to *R*_1_ (3.43% ± 1.15%), *t*(10) = −4.78, *p* = 0.0007, Cohen's *d* = −1.44. Similarly, CV for the VL muscle IMPI was increased in *R*_2_ (6.49% ± 2.01%) relative to *R*_1_ (4.09% ± 0.86%), *t*(10) = −4.94, *p* = 0.0005, Cohen's *d* = −1.49 (Figure 3). No significant differences (*p* = 0.074) in the CV were detected for the GM muscle IMPI between *R*_1_ (5.19% ± 1.23%) and *R*_2_ (6.67% ± 2.21%).

**FIGURE 4:**
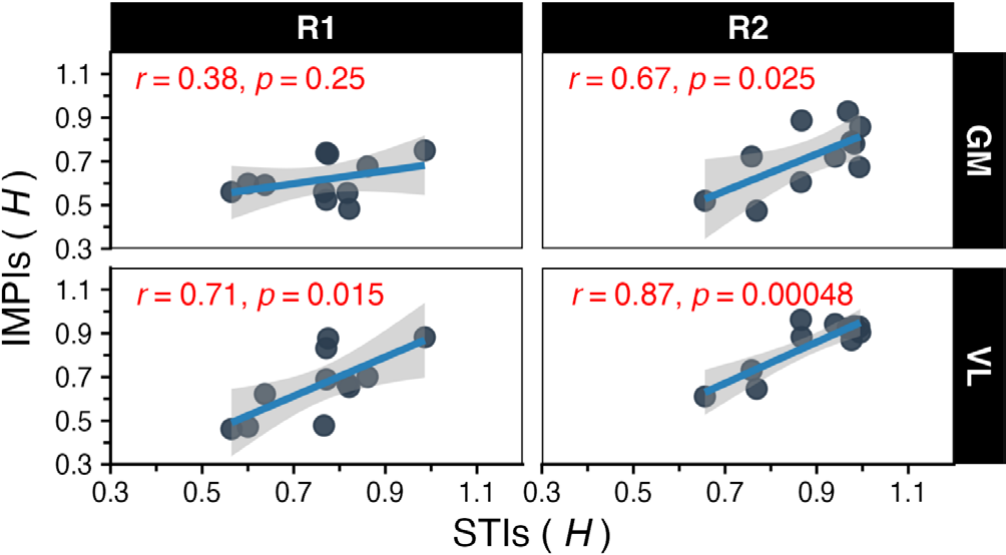
The correlation between Inter‐Muscle Activity Intervals (IMPI) and Inter‐Stride Intervals (STI) H‐exponents for each of the test runs. The individual data points represent a unique participant value.

The results of the variability matching for Hurst exponent values showed a moderate (*r* = 0.38, *p* = 0.25) and insignificant correlation between STI and the IMPI of the GM muscle for *R*_1_ and a statistically significant moderate to strong correlation (*r* = 0.67, *p* = 0.025) for *R*_2_. For the VL muscle, a strong and statistically significant correlation (*r* = 0.71, *p* = 0.015) was observed in *R*_1_ and this pattern got stronger (*r* = 0.87, *p* = 0.00048) in *R*_2_, as depicted in Figure 4.

**FIGURE 5:**
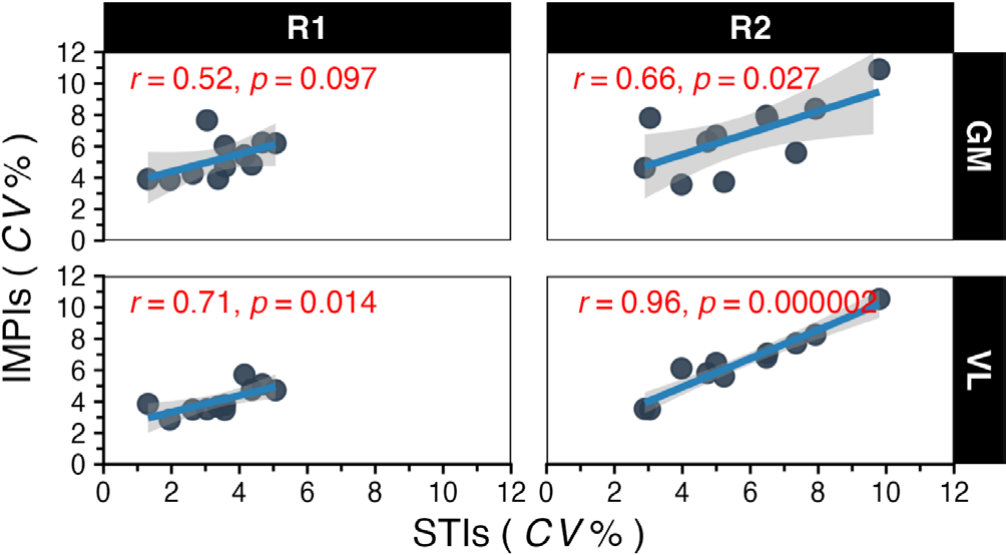
The correlation between Inter‐Muscle Activity Intervals (IMPI) and Inter‐Stride Intervals (STI) CV values for each of the test runs. The individual data points represent a unique participant value.

Regarding the correlations involving the CV, analysis of the GM muscle revealed a moderate, non‐significant relationship between STI and IMPI during *R*_1_ (*r* = 0.52, *p* = 0.097). This correlation increased and became statistically significant during *R*_2_ (*r* = 0.66, *p* = 0.027). For the VL muscle, a strong and significant correlation was already evident during *R*_1_ (*r* = 0.71, *p* = 0.014), and this relationship intensified to a very strong, highly significant correlation during *R*_2_ (*r* = 0.96, *p* = 0.000002), as depicted in Figure 5.

## 4 Discussion

The purpose of the present paper was to investigate the effect of fatigue on STI and IMPI temporal structure and magnitude of variability as well as on their association in trained track and field athletes. Specifically, we investigated whether introducing higher physical demands in two consecutive (*R*_1_, *R*_2_) submaximal (400‐m) running trials would induce changes in the Hurst exponents and CV values of STI and IMPI as well as the correlation between the two. As a preliminary hypothesis, we tested whether the protocol effectively induced fatigue, expecting lower baseline muscle oxygenation (SmO_2_) before *R*_2_ compared with *R*_1_, accompanied by distinct neuromuscular alterations—specifically, an increase in EMG signal amplitude (RMS) and a concurrent decrease in the spectral characteristics (MDF). We also hypothesized that the second run (*R*_2_), performed under residual fatigue, would show reduced persistency and increased magnitude of variability compared to the first run (*R*_1_). Finally, we expected that the relationship between STI and IMPI (both in terms of the structure and magnitude of variability) would be stronger in *R*_2_, reflecting increased coupling between subsystems under fatigue.

Our preliminary hypothesis was supported by both metabolic and neuromuscular markers: baseline muscle oxygenation was decreased prior to *R*_2_ compared to *R*_1_, while EMG activity exhibited the characteristic fatigue response of increased amplitude accompanied by decreased median frequency. This physiological marker validates that the experimental protocol effectively induced residual fatigue between the two runs. Crucially, Tomazin et al. [43] demonstrated that even a single 400 m sprint is sufficient to induce a 14% drop in maximal voluntary contraction. By utilizing a dual‐bout protocol with incomplete recovery, our study imposed a physiological load exceeding this established fatigue threshold. Contrary to our first hypothesis, Hurst exponents for both STI and IMPI were higher in *R*_2_ than in *R*_1_, suggesting that complexity under fatigue (at least for the specific task) does not follow the results of the literature [20, 21, 22, 23], which, though, does not provide data from comparable running tests. The rest of our hypotheses were verified as the CV increased from *R*_1_ to *R*_2_ for both STI and IMPI, and the correlation coefficients were increased for both CV and Hurst exponents.

The significant increase in the STI Hurst exponents during fatigued running contradicts previous findings. Prior research indicates that the variability in STI demonstrates an optimal structure that appears to diminish under conditions of fatigue [20, 21, 22, 23]. However, these experiments estimated the Hurst exponent using the DFA algorithm in prolonged running trials. In these long efforts, fatigue appears to produce variability patterns that are less complex and more random. These reference data are in line with evidence suggesting that kinematic alterations can be attributed to the increased physiological demands during running [44]. However, our results do not support this proposition, despite the accumulation of fatigue byproducts that is reflected in increased saturation of muscle oxygenation [45, 46] and increased magnitude of variability [21, 23]. We believe that the EMG extracted features and described above provide a physiological basis for this shift towards increased persistency. The protocol successfully induced significant neuromuscular fatigue, evidenced by the classic signatures of a decrease in MDF in both the VL and GM and a compensatory increase in signal amplitude (RMS) in both muscles [30, 31, 32]. This indicates that fatigued muscle fibers were slowing their firing rates, forcing the central nervous system to increase neural drive to maintain performance. Crucially, this fatigued state was also accompanied by a significant change in the GM's IQR—MDF distribution. This suggests that the neuromuscular system adopted a more rigid and stereotyped control strategy, with less variability in its firing frequency from burst to burst. This emergent “rigidity” at the muscular level offers a possible explanation for the increase in the Hurst exponent for both STI and IMPI. This narrowing of the neuromuscular repertoire (indicated by the IQR‐MDF changes) implies that the central nervous system has fewer effective motor solutions available. Consequently, the capacity to decouple muscle activation from stride mechanics is diminished. The system effectively ‘locks’ the gait cycle to the neural drive to preserve output, resulting in the tighter coupling and reduced flexibility observed. An increase in Hurst signifies greater persistency—a state where the system is less flexible and more “locked in” to its current dynamics. Therefore, under this specific high‐intensity task, the body's response was not to break down towards randomness (as often seen in prolonged, aerobic fatigue), but rather to adopt a more persistent, and less flexible motor pattern as a strategy to cope with the acute fatigue. This fundamental difference suggests that while prolonged running likely leads to a ‘loosening’ of control (lower Hurst), acute high‐intensity fatigue forces the system into a protective state of ‘neuromuscular rigidity’ (higher Hurst). Interestingly, while the temporal structure (Hurst exponent) responded differently than expected, the increase in CV with fatigue is consistent with prior literature on linear gait variability [28, 29]. This result suggests that linear indicators of variability, such as CV, may reflect general fatigue‐related disruption, regardless of duration or intensity. In contrast, nonlinear dynamics appear to be more sensitive to the specific type or profile of fatigue, with short‐term high‐intensity efforts eliciting different adaptations than prolonged exertion.

Our results suggest that such structure, usually observed in gait patterns, is also present in muscular activation. Similar to previous findings, the correlation between IMPI and STI Hurst exponents appears to be strong [5, 6]. However, a stronger correlation was found for *R*_2_ compared to *R*_1_. This was strongly reflected in correlation coefficients of 0.87 for *R*_2_ and 0.71 for *R*_1_ for VL IMPI and STI. This implies that the examined subsystems contributing to the running outcome operate more independently in the absence of fatigue. Our results therefore support our third hypothesis, demonstrating that when the locomotor system operates within an optimal state, its subcomponents can fluctuate independently across multiple time scales. Prior research has examined the integration of various subsystems during gait. Coupling between both cardiac and locomotor and also neuromuscular and locomotor systems has been observed during walking [5, 6, 10, 13]. Our observation that fatigue strengthens the complexity matching between subsystems aligns also with recent findings in coordination dynamics, suggesting this may be a general property of biological systems under stress. For instance, De Jonge‐Hoekstra et al. [47] reported that increasing task difficulty led to stronger complexity matching between speech and gestures. They proposed that this tighter coupling serves as a stabilization mechanism when the system is perturbed or challenged. Similarly, in our study, the shift from the ‘easy’ (fresh) condition of *R*_1_ to the ‘difficult’ (fatigued) condition of *R*_2_ forced the locomotor and neuromuscular systems to abandon their independent fluctuations. Instead, they adopted a highly coupled, mutually constrained behavior—potentially to preserve motor output stability despite the degrading physiological capacity. Moreover, the correlation was increased in *R*_2_ for the CV as well. This result may suggest that the magnitude of variability follows the same trend as the temporal structure among subsystems.

Few studies have examined the matching of complexity across multiple systems. Specifically, the increase in CV alongside divergent responses in the Hurst exponent suggests that short‐term fatigue alters motor complexity differently than prolonged fatigue, pointing to the need for fatigue‐type‐specific monitoring strategies. Increasing matching can be important for rehabilitation too, as it could enhance interventions targeting multiple physiological systems. For example, external visual cueing has been shown to alter the temporal structure of STI and induce parallel changes in IMPI and R‐R intervals, suggesting cross‐system adaptability [6, 10].

We believe the findings of this study offer some direct applications for sport practitioners and monitoring. First, we demonstrated that the Hurst‐Kolmogorov (HK) algorithm is a viable tool for assessing gait complexity in short‐duration maximal efforts, such as the 400‐m sprint, where traditional methods (e.g., DFA) are often inapplicable due to data length constraints. Second, the divergent behavior of linear (CV) and non‐linear (Hurst) metrics suggests that a single variability metric is insufficient; practitioners should monitor both to distinguish between general performance disruption and specific fatigue‐induced rigidity. Finally, the observed increase in coupling between muscle activity and stride patterns serves as a potential biomarker for neuromuscular “tightening,” which could help identifying the threshold where training stress transitions into maladaptive fatigue. To our knowledge, the present study is the first to examine how high‐intensity running influences the complexity in stride‐to‐stride variability and the matching of cause (EMG) and effect (stride intervals). As such, several considerations arise. First, there are no established Hurst exponent values for a 400‐m sprint, so we cannot yet determine whether our observed Hurst exponent values exceed the normative range. Although the rise in CV according to the literature could imply increases in fatigue [23, 28], two maximal efforts may have been insufficient to push the locomotor system to its true fatigue threshold. In that case, the increase in Hurst exponents may instead reflect an adaptive increase in complexity that is accompanied by increased coupling. Future research should investigate the existence of a threshold during short‐term high‐intensity training after which changes in the structure of variability become detrimental to the adaptive capacity of the individual. If such a case exists, then the metrics of complexity and their correlations between different subsystems may become a training guide regarding the duration and limits of training. As interest grows in restoring complexity in human variability, interventions targeting multiple systems could potentially entrain both gait and broader physiological rhythms.

The implications that our results suggest could be further strengthened if the following limitations are addressed in future experiments. Although the technique we used to compute the Hurst exponent is validated and reliable, the comparison of our results with similar research should be done with caution. Conducting further research into short‐duration running tasks could offer a deeper understanding of the complexity of the locomotor system in such highly demanding tasks, especially if more repetitions are used. Finally, while bipolar EMG provided macro‐level insights into neuromuscular fatigue, it limits our ability to identify specific motor unit behaviors. Future research should employ high‐density EMG arrays and signal decomposition techniques [48] to isolate constituent motor unit action potential trains and verify how recruitment strategies contribute to the observed loss of complexity.

## 5 Conclusion

This study provides preliminary evidence of coupling between stride time and muscle activation intervals. We conclude that under non‐fatigued baseline conditions, these two subsystems of human locomotion exhibit a certain relationship, which strengthens as fatigue progresses. Further experiments are necessary to determine whether greater fatigue (often linked to reductions in complexity) is associated with stronger relationships or, alternatively, whether a breakdown in the interdependence of the subsystems emerges.

## 6 Perspectives

The findings of this study contribute to neuromuscular control theory by demonstrating that acute high‐intensity fatigue induces a state of “neuromuscular rigidity” rather than the increased randomness often reported in prolonged aerobic exercise. From a sports science perspective, these results identify the increased matching of subsystem complexities as a potential biomarker for monitoring training load and athlete readiness. With application of the Bayesian Hurst‐Kolmogorov method for short‐duration tasks, we provide practitioners with a viable tool to assess movement complexity during high‐intensity intervals where traditional analytical methods typically fail. Distinguishing between general performance decline and structural rigidity allows for more nuanced monitoring of maladaptive fatigue, potentially refining training periodization and injury prevention strategies.

## Author Contributions

All authors read and approved the final version of the manuscript.

## Funding

The authors have nothing to report.

## Conflicts of Interest

The authors declare no conflicts of interest.

## Data Availability

The data that support the findings of this study are available from the corresponding author upon reasonable request.
